# *Notes From the Field*: Severe Illnesses After Self-Injection of Botulinum Toxin Purchased Online — New York, Texas, and Wisconsin, 2025

**DOI:** 10.15585/mmwr.mm7438a1

**Published:** 2025-11-27

**Authors:** Liz Lamere, Jennifer Cope, Robert Breazu, Sandra Peña, Michelle Chang, Joel Ackelsberg, Divya Pillendla, Olivia A. Smith, Quoc Phung Than, Pilar Zaibaq, Suzanne Gibbons-Burgener, Ryan J. Wozniak, Ethel Taylor

**Affiliations:** ^1^Division of Foodborne, Waterborne, and Environmental Diseases, National Center for Emerging and Zoonotic Infectious Diseases, CDC; ^2^Epidemic Intelligence Service, CDC; ^3^New York City Department of Health and Mental Hygiene, New York, New York; ^4^Harris County Public Health, Houston, Texas; ^5^Texas Department of State Health Services; ^6^Wisconsin Department of Health Services.

**Summary**

**What is already known about this topic?**

Injection of botulinum neurotoxin (BoNT) approved by the Food and Drug Administration (FDA) for therapeutic and cosmetic purposes is considered safe when administered by a licensed and trained medical professional.

**What is added by this report?**

During May–June 2025, three persons experienced severe illness requiring hospitalization after self-injecting BoNT purchased online. After their clinical teams consulted with CDC, all three patients received botulinum antitoxin and recovered with varying residual signs and symptoms.

**What are the implications for public health practice?**

BoNT should only be administered by licensed and trained medical professionals using recommended doses of FDA-approved products purchased directly from the manufacturer or through authorized distributors. Self-injection of BoNT purchased online from unauthorized sources can result in serious adverse health effects.

Cosmetic botulinum neurotoxin (BoNT) can be used to temporarily diminish facial wrinkles (*1*); however, injection for this purpose occasionally results in localized paralytic effects, even when BoNT that is approved by the Food and Drug Administration (FDA) and purchased from authorized sources is administered by licensed and trained medical professionals. Rarely, improperly procured or administered BoNT can lead to severe illness. During May–June 2025, hospital clinicians and health departments in New York, Texas, and Wisconsin each alerted CDC about a person in their jurisdiction who experienced severe illness after self-injecting cosmetic BoNT that was purchased online.* None of the three patients met their state’s requirements for purchasing or administering BoNT; no link was reported among the patients. This report describes the patients’ characteristics, treatment, and outcomes. This activity was reviewed by CDC, deemed not research, and conducted consistent with applicable federal law and CDC policy.^†^

## Investigation and Outcomes

### Patients Reported to CDC

During May–June 2025, three women aged 29, 50, and 59 years (one each from New York, Texas, and Wisconsin, respectively) self-injected BoNT and subsequently experienced severe illness (Table). One patient injected BoNT into her face, one into her neck, and the third into her neck and face. Reported total units that were self-administered ranged from 11 to approximately 200. None of the patients was reported to have a health care license, be a professional injector, or otherwise have the training and credentials required to purchase or inject BoNT; no link was reported among the patients. CDC was notified about the patients by their respective jurisdictional health departments.

**TABLE:** Characteristics of patients who self-injected botulinum toxin purchased online — New York, Texas, and Wisconsin, May–June 2025

Characteristic	Patient A	Patient B	Patient C
Injection site	Eyebrows Forehead Lips	Platysmal bands	Cheeks Eyebrows Forehead Masseters Platysmal bands Trapezius
Injection dose (as reported by patient)	11 units	Approximately 200 units	Approximately 200 units
No. of days from injection to first symptom	4	2	3
Clinical features*	Bilateral upper extremity weakness Diplopia Dysphagia Dysphonia Unilateral ptosis	Bilateral upper extremity weakness Dysarthria Dysphagia Orthopnea Reduced oral intake Shortness of breath	Facial, neck, and extremity weakness Dysarthria Dysphagia Increased work of breathing Ptosis
Required invasive mechanical ventilation	No	Yes	No
No. of days hospitalized	3	6	4
Source of product (location)	Online retailer (Korea)	Vendor on a messaging platform (WhatsApp) (China)	Vendor on a social media platform (TikTok) that communicated via a messaging platform (WhatsApp) (China)
Laboratory method and result	MALDI-TOF-MS; serum negative for BoNT	Mouse bioassay; serum negative for BoNT	MALDI-TOF-MS; serum negative for BoNT
Residual morbidity at discharge	None	Some residual dysphagia	Dysarthria Neck weakness Ptosis

**Abbreviations:** BoNT = botulinum neurotoxin; MALDI-TOF-MS = matrix-assisted laser desorption/ionization time-of-flight mass spectrometry.

* Electromyography (which can be used to aid in the diagnosis of botulism) was not conducted for any patient.

### Clinical Complications and Course of Illness

Illness onset occurred 3–5 days after injection and included dysphagia, dysarthria, diplopia, ptosis, respiratory difficulties, upper extremity weakness, and other signs and symptoms (Table). Each patient was evaluated in an emergency department because of symptom progression. All three were hospitalized (with length of hospitalization ranging from 3 to 6 days). One patient required invasive mechanical ventilation because of airway concerns. After the clinical teams for the three patients learned about the history of BoNT self-injection from their patients, all suspected botulism. They coordinated with their respective jurisdictional health departments to contact CDC’s clinical botulism service about the potential use of botulism antitoxin (BAT), an equine-derived preparation of antibodies that bind to and neutralize BoNT in the bloodstream that has not yet irreversibly bound to synaptic receptors to prevent progression of paralysis and consequent complications (*2*).

### Consultation with CDC and Release of Botulism Antitoxin

Following these separate consultations, each clinical team requested heptavalent BAT, which was released by CDC.^§^ Before administration, a blood specimen was collected from each patient and shipped to public health laboratories to test for the presence of BoNT. Per CDC botulism clinical guidelines, BAT treatment was initiated based on clinician suspicion of botulism, before BoNT testing results were available (*2*). BAT was administered to each patient 3–4 days after symptom onset. All patients were discharged from the hospital with varying degrees of residual morbidity. BoNT testing results were available after discharge; circulating BoNT was not detected in the serum of any of the three patients.

### Efforts to Identify Source of BoNT

Health department staff members attempted to identify the source of the products. Patients provided limited product information and reported that the products were purchased online from sources outside the United States. Some patients communicated with vendors via messaging platforms such as WhatsApp. None provided direct online links to the products or vendors, and health departments were unable to obtain any of the products for testing. One patient noted using the internet to research injection techniques for specific anatomic sites. Additional follow-up to identify product vendors was unsuccessful. 

## Preliminary Conclusions and Actions

Licensed medical providers have safely administered FDA-approved BoNT for decades, despite its being one of the most potent naturally occurring toxins (*2**,**3*). However, unsafe use (e.g., use of counterfeit products, administration by unlicensed providers, or self-injection) has resulted in severe illnesses, with some patients requiring invasive mechanical ventilation and administration of BAT; no deaths have been reported (CDC, unpublished data, 2025). Health care providers should counsel persons interested in BoNT injections about risks associated with improper administration and encourage them to seek injections only from licensed, trained medical professionals operating in accordance with jurisdictional requirements and using FDA-approved BoNT. Misleading and inappropriate guidance from social media regarding self-injection of BoNT should be countered with information about the associated risks.

The illnesses described in this report are consistent with local and possible remote anatomic spread of BoNT, similar to previously reported cases that occurred after cosmetic injections of counterfeit or presumed counterfeit BoNT products (*4*). BoNT was not detected in the serum of any of the three patients, a common finding in all types of botulism (*2**)*. Possible reasons BoNT was not detected in these patients include 1) BoNT did not circulate in these patients’ bloodstreams, and their clinical features were the result of diffusion, rather than circulatory dissemination, of BoNT from where it was injected to nearby muscles; 2) BoNT was circulating in the patients’ bloodstream when the specimens were obtained but at levels below the assay detection threshold; or 3) BoNT that previously circulated in the patients’ bloodstream had already bound to synaptic receptors when the specimens were obtained. The 3-day to 4-day delay between symptom onset and collection of serum specimens could have precluded detection of BoNT that had previously been present. BoNT has been detected rarely in serum after cosmetic or therapeutic injection, with fewer than 10 such cases identified in the United States (CDC, unpublished data, 2025). It is unclear how beneficial BAT administration was in these three patients because there was no detectable BoNT for the BAT to bind to at the time of administration. The limited information provided by the patients about the products they used suggests that none were FDA-approved products; notably, all three patients reported purchasing BoNT online from vendors in Asia.

Medications, including BoNT, that are purchased from unlicensed sources such as online retailers might be misbranded, mislabeled or unlabeled, adulterated, counterfeit, contaminated, improperly stored or transported, ineffective, or unsafe^¶^ (*5*). Persons without a health care license should not purchase BoNT online and should only receive injections from a provider authorized to purchase and administer BoNT in their state. Although requirements for BoNT injection vary by state, patients can check with their local licensing boards or locate authorized providers through provider lookup tools available on FDA-approved BoNT product or professional organization websites. Health care providers should avoid purchasing BoNT from unlicensed sources. Warning signs that online pharmacies might be selling unsafe products include having an address outside the United States or selling damaged, mislabeled, expired, or deeply discounted products (FDA | Considering an Online Pharmacy?).

CDC offers a clinical botulism service (telephone: 770-488-7100) 24 hours a day to provide information for health care providers and health departments about diagnosing botulism and using BAT. FDA’s MedWatch program is available for reporting adverse effects from BoNT products, as well as possible counterfeit BoNT products (FDA | MedWatch Online Voluntary Reporting Form 3500).

## Footnotes

* Requirements for the legal purchase and administration of BoNT vary by state and often include having a valid medical, nursing, or dental license (or working under the delegation and supervision of a licensed medical professional such as a medical doctor) and being trained to administer BoNT. The recommended BoNT treatment dose (number of units) differs depending on the treatment area and product used, with FDA-approved BoNT products ranging from 20 to 50 units per cosmetic indication.

† 45 C.F.R. part 46, 21 C.F.R. part 56; 42 U.S.C. Sect. 241(d); 5 U.S.C. Sect. 552a; 44 U.S.C. Sect. 3501 et seq.

§ In the United States, heptavalent BAT, which is most effective 24–48 hours after onset of neurologic symptoms, is requested when a patient is suspected to have botulism. Each single-use vial contains a minimum potency of 4,500 units (for serotype A antitoxin), 3,300 units (B antitoxin), 3,000 units (C antitoxin), 600 units (D antitoxin), 5,100 units (E antitoxin), 3,000 units (F antitoxin), and 600 units (G antitoxin). The U.S. Department of Health and Human Services maintains the antitoxin supply, and CDC has processes for releasing doses in coordination with jurisdictional health departments. BAT is only effective when circulating BoNT is present; testing and treatment are initiated simultaneously due to the lengthy confirmatory testing process. BoNT testing is performed via matrix-assisted laser desorption/ionization time-of-flight mass spectrometry or mouse bioassay.

¶ Obtaining BoNT products from unregulated suppliers, many of which are online suppliers outside the United States, can result in patients receiving a product of unknown quality and safety. Consequently, patients could receive an injection containing dangerously potent BoNT, as well as additional ingredients that could lead to local and systemic reactions or toxicities.
